# Tardive Dyskinesia Development, Superoxide Dismutase Levels, and Relevant Genetic Polymorphisms

**DOI:** 10.1155/2022/5748924

**Published:** 2022-10-28

**Authors:** Kadir Uludag, Dong Mei Wang, Xiang Yang Zhang

**Affiliations:** CAS Key Laboratory of Mental Health, Institute of Psychology, Chinese Academy of Sciences, Beijing, China

## Abstract

Tardive dyskinesia (TD) is a prevalent movement disorder that significantly impacts patients with schizophrenia (SCZ) due to extended exposure to antipsychotics (AP). Several genetic polymorphisms, including superoxide dismutase (SOD) and DRD3 9ser, have been suggested as explanations why some patients suffer from TD. *Methods*. A PubMed search was used to search relevant articles using the following keywords: “Tardive Dyskinesia and Superoxide Dismutase”. Fifty-eight articles were retrieved. Among them, 16 were included in this review. *Results*. Overall, 58 studies were retrieved from PubMed. Most studies investigated the association between TD and the SOD-related polymorphisms. In addition, previous studies reported an association between TD occurrence and other genetic polymorphisms. *Conclusion*. This study found that the risk of TD is associated with altered SOD levels and several genetic polymorphisms, including VAL 66 Met and DRD3 9ser.

## 1. Introduction

Tardive dyskinesia (TD) is a movement disorder that occurs due to an excessive exposure to antipsychotics (AP), an essential problem in schizophrenia (SCZ) patients since they need to use APs for an extended period [1]. However, it is unclear why some patients develop TD, while some do not, and therefore, genetic studies have been done to try to account for the susceptibility to TD [2]. The superoxide dismutase (SOD) polymorphism is one of the candidates reported to be related to TD occurrence. SODs are universal enzymes [3] that have an essential role in inflammatory diseases [4] and oxidative stress (OS) [5]. Therefore, due to their relation to these diseases, they are associated with cognitive functions such as memory and attention.

Many previous studies have found an association between serum SOD levels and TD occurrence [6, 7]. Additionally, SCZ patients with TD have lower copper-zinc couple SOD (CuZnSOD) activity than non-TD patients [8]. Similarly, a different study found a decrease in erythrocyte SOD activity in the TD group than the non-TD group [9].

Recently, genetic studies concerning TD susceptibility have gained importance, and the combination of the MnSOD-9val and DRD3 9ser alleles has been associated with TD (Z. J. [10]). Furthermore, a study found that the excess of the -697 variant in the promoter regulation of the *HTR2C* gene may be a risk factor for TD [10, 11]. However, a different study did not support the previous findings and reported that the *MnSOD* gene Ala-9Val polymorphism did not have a role in TD risk [12].

It is crucial to consider that the previous studies may have adopted different methods to investigate the association between SOD and TD, and taken together, systematically exploring the association between TD and SOD-related parameters is vital. Furthermore, we have investigated the association between TD and SOD-related parameters according to inflammation and OS concerning cognitive deficits.

## 2. Methods

A PubMed search was used to find articles relevant to tardive dyskinesia (TD) and superoxide dismutase (SOD) using the keywords “tardive dyskinesia and superoxide dismutase”. Overall, 58 articles were found. Animal studies were excluded from this study, as well as non-English articles, articles prior to 1997, and articles not relevant to this research question. All the included studies were written in English and published between 1997 and 10 June 2022 (total of 16 studies were included).

SOD activity is not simply observed directly due to the rapid substrate disappearance at physiological pH [13]. SOD levels can be measured in several ways, including in serum and erythrocytes. For most of the studies mentioned in this review, determination of plasma total SOD activities was executed using an assay involving spectrophotometric determination as in previous study [14]. In addition, genotyping was performed by polymerase chain reaction (PCR). For some studies, the genotypes were combined by their functional significance to investigate the interaction between polymorphisms.

There are no well-established and comprehensive methods for measuring OS in SCZ patients [15]. Overall, the techniques for measuring OS include reactive oxygen species (ROS) fingerprinting and ROS in body fluids [16]. In addition, OS can be investigated in the peripheral and central nervous systems. Moreover, regional variations in OS in human skin can be observed [17]. However, it is essential to note that antioxidant capacity markers are suggested to be associated with oxidative damage markers, although they are insufficient [18]. Several methods have been used to investigate oxidative base modifications, including gas and liquid chromatography [19]. Additionally, developing technology has helped to determine the location of oxidation-related DNA damage [19].

## 3. Results

A total of 16 articles were retrieved related to tardive dyskinesia (TD) and superoxide dismutase (SOD) and included in this study (Table 1). In addition, a few articles were also related to cognitive skills and TD.

### 3.1. Results of Studies Related to Superoxide Dismutase-Related Polymorphisms

The included studies investigated the association between TD and Ala-9Val, DRD3 9ser, NQO1 Pro187Ser, and HTR2C polymorphisms. Furthermore, the studies investigated the combination of genes, including the combination of the MnSOD -9val and CAT-262C>T, and DRD3 9ser alleles were associated with TD [10, 11]. The combined genotypes of T/T in NQO1 Pro187Ser and Val/Val in MnSOD Ala-9Val polymorphisms were associated with a higher TD risk [20]. In addition, the excess of the -697 variant in the promoter regulation of the *HTR2C* gene may be a risk factor for TD [10, 11]. Moreover, no critical roles of SOD2Val16Ala, CAT-262C>T, and GPX1Pro200Leu polymorphisms in TD occurrence were found [21].

### 3.2. Results of Studies Related to Serum Superoxide Dismutase Levels

Several studies investigated the relationship between SOD serum levels and TD occurrence.

One study showed lower CuZnSOD activity in patients with TD than those without TD [6, 7]. Furthermore, SCZ patients with TD had lower CuZnSOD activity than patients without TD [8].

Another study showed that MnSOD activity was lower in patients with TD than in non-TD patients [22]. In addition, a different study showed a decrease in erythrocyte SOD activity in the TD group compared with the non-TD group [9].

## 4. Discussion

This study found that superoxide dismutase (SOD) is vital in understanding the risk of TD occurrence and may be used as a biomarker to predict TD occurrence. Moreover, most of the studies related to genetics were focused on the SOD-related polymorphisms.

Despite the common literature, the papers were difficult to compare due to the differences in study samples, including age, study design, comorbid disorders, and hospitalization status; the aim of the studies; and the methodologies.

SOD regulates OS, inflammation, and oxidation [23]. Some environmental factors may also be associated with MnSOD, such as exercise [24]. In addition, some studies mentioned the link between SOD-related parameters and cognitive skills.

TD-related theories focused on postsynaptic D2 receptor supersensitivity and dopamine hyperactivity [25] while D2 receptor hypersensitivity and degenerative changes in the neurons due to exposure to oxidative stress (OS) can lead to impacts on the synaptic plasticity of glutamatergic synapses, leading to an imbalance between pathways of basal ganglia and creating aberrant output to the sensorimotor cortex [26, 27]. Therefore, OS-related parameters may be used to understand TD mechanisms.

### 4.1. The Relationship between Superoxide Dismutase (SOD) and Oxidative Stress

Superoxide is a prevalent ROS produced by the mitochondria [28]. SODs are essential in treating OS-related diseases [29], and elevated SOD was related to lower mortality in older females [30]. A balance in ROS is essential, and SOD, a normal metabolite in standard amounts, facilitates critical roles [5]. The structure and location of SODs are vital for a healthy balance of superoxides [31]. Therefore, therapeutics targeting oxidation and superoxides should be investigated for treating neurodegeneration [32].

### 4.2. The Relationship between Superoxide Dismutase (SOD) and Cognitive Skills

Previous studies have investigated the association between SOD and specific cognitive functions and have found that extracellular SOD played a vital role in various cognitive functions [33]. Another study demonstrated the role of reactive oxygen species (ROS) in learning deficit (R. [34]).

Studies with animal models that overexpressed superoxide scavengers demonstrated that some neuronal processes are changed during diminished superoxide-related signaling [35]. Furthermore, a different study suggested that participants with late-life SCZ had disturbances in their antioxidant system, which was related to cognitive problems [36]. Therefore, an antioxidant with mitochondrial activity may improve cognitive impairments [37]. One study investigated changes in SOD levels after antioxidant kaempferol use and demonstrated increased SOD and diminished memory problems in rats [38]. Moreover, it has also been found that low SOD was related to an increased risk of poststroke cognitive deficiency (M.-S. [39]).

Taken together, SOD may play an essential role in cognitive skills, and antioxidants may relieve TD symptoms.

### 4.3. Antioxidant Treatment on Relieving Tardive Dyskinesia Symptoms

Some antioxidants, including Ginkgo biloba, vitamin E, omega 3, piracetam, and curcumin, could reduce the severity of TD symptoms ([40]; X. Y. [41]). Elsewhere, it has been found that resveratrol enhanced the expression of antioxidant enzymes such as heme oxygenase 1 and SOD, which are responsible for redox balance [42]. Consequently, antioxidants have been commonly suggested to possibly alleviate the severity of TD symptoms through oxidative mechanisms.

## 5. Conclusion

In conclusion, SOD levels and several SOD polymorphisms (e.g., MnSOD -9val and DRD3 9ser) are vital in understanding the risk of TD. Also, some SOD polymorphisms may be related to the severity of TD symptoms.

## 6. Limitations

This study had some limitations. Firstly, our review does not include all the studies that were related to SOD. Additionally, the study did not apply meta-analysis methods in analyzing the data.

## 7. Suggestions for Further Studies

Future research should use a meta-analysis to further investigate the association between TD and SOD. Additional studies should demonstrate the treatment response of antioxidants using genetic methods.

## Figures and Tables

**Table 1:** Studies found using PubMed.

Author of study	Title of study	Goal of study	Results of study
(1) Wu et al. [7]	Association of altered CuZn superoxide dismutase and cognitive impairment in schizophrenia patients with tardive dyskinesia	Investigate the activity of CuZnSOD	There is reduced CuZnSOD activity in TD patients compared with patients without TD.
(2) Wu et al. [22]	Mn-superoxide dismutase activity is associated with orofacial involuntary movements in schizophrenia patients with tardive dyskinesia	Investigate the role of OS in SCZ patients with TD	MnSOD activity was lower in TD patients than in non-TD patients.
(3) Zhang et al. [10]	Interaction between polymorphisms of the dopamine D3 receptor and manganese superoxide dismutase genes in susceptibility to tardive dyskinesia	The impact of a polymorphism in the dopamine D3 receptor and its interaction with MnSOD in TD patients	The combination of the MnSOD -9val and DRD3 9ser alleles was associated with TD.
(4) Wu et al. [6]	Cognition impairment in schizophrenia patients with tardive dyskinesia: association with plasma superoxide dismutase activity	The function of OS in TD patients and the cognitive problems associated with MnSOD	Patients with TD had a lower RBANS score than the non-TD group.
(5) Liu et al. [43]	Association of the manganese superoxide dismutase gene Ala-9Val polymorphism with clinical phenotypes and tardive dyskinesia in schizophrenic patients	If the *MnSOD* gene and the Ala-9Val polymorphism was associated with AP-induced TD	The *MnSOD* gene with the Ala-9Val polymorphism was not associated with TD.
(6) Zhang et al. [44]	The effect of vitamin E treatment on tardive dyskinesia and blood superoxide dismutase: a double-blind placebo-controlled trial	The effect of vitamin E on SOD	There was a greater reduction in the AIMS score with vitamin E treatment compared with the placebo. Blood SOD levels were increased after treatment with vitamin E.
(7) Su et al. [45]	Relationship between tardive dyskinesia and the polymorphism of superoxide dismutase val9Ala and efficacy of Chaihu Taoren capsules on it	The gene distribution rate of Val9Ala gene was analyzed and the therapeutic effect of CTD was analyzed on patients with TD	There was no difference in allelic gene frequency of SOD Val9Ala among the groups (TD, without TD).
(8) Pae [20]	Additive effect between quinine oxidoreductase gene (NQO1: Pro187Ser) and manganese superoxide dismutase gene (MnSOD: Ala-9Val) polymorphisms on tardive dyskinesia in patients with schizophrenia	Whether there is an interaction between the NQO1 Pro187Ser and MnSOD Ala-9Val gene polymorphisms in TD	The combined genotypes of T/T in NQO1 Pro187Ser and Val/Val in MnSOD Ala-9Val polymorphisms were associated with a higher TD risk.
(9) Tsai et al. [46]	Markers of glutamatergic neurotransmission and oxidative stress associated with tardive dyskinesia	To study if neuroleptics enhance striatal glutamatergic neurotransmission via blocking the presynaptic dopamine receptors	TD patients had higher concentrations of N-acetylaspartate, N-acetylaspartylglutamate, and aspartate in their CSF compared with patients without TD.
(10) Yamada et al. [9]	Low superoxide dismutase activity in schizophrenic patients with tardive dyskinesia	To analyze the association between erythrocyte SOD activity and TD	There was a decrease in erythrocyte SOD activity in the TD group compared with the non-TD group.
(11) Gałecki et al. [8]	Pro- and antioxidant processes in schizophrenics with tardive dyskinesia	An assessment of SOD, catalase, glutathione peroxidase activity, and lipid peroxidation in blood platelets of patients with or without TD	SCZ patients with TD had lower CuZnSOD activity than patients without TD.
(12) Zhang et al. [11]	Pharmacogenetic assessment of antipsychotic-induced tardive dyskinesia: contribution of 5-hydroxytryptamine 2C receptor gene and of a combination of dopamine D3 variant allele (Gly) and MnSOD wild allele (Val)	Whether the functional polymorphisms in the dopamine D2 receptor and dopamine D3 receptor genes associated with TD	The excess of the -697 variant in the promoter regulation of the *HTR2C* gene may be a risk factor for TD.
(13) Hitzeroth et al. [47]	Association between the MnSOD Ala-9Val polymorphism and development of schizophrenia and abnormal involuntary movements in the Xhosa population	Whether the polymorphism (Ala-9Val) in the *MnSOD* gene was associated with SCZ	*MnSOD* Ala-9Val polymorphism may modulate the severity of TD.
(14) Al Hadithy et al. [48]	Missense polymorphisms in three oxidative-stress enzymes (GSTP1, SOD2, and GPX1) and dyskinesias in Russian psychiatric inpatients from Siberia	To examine the relationship of TD and OS	An association between MnSOD Ala-9Val and TD orofaciolingual was found.
(15) Bošković et al. [21]	Association of SOD2, GPX1, CAT, and TNF genetic polymorphisms with oxidative stress, neurochemistry, psychopathology, and extrapyramidal symptoms in schizophrenia	To evaluate polymorphisms of Mn SOD2Val16Ala, glutathione peroxidase GPX1Pro200Leu, catalase CAT-262C>T, CATc.66+78C>T, and TNF-alpha TNF-308G>A by assessing their association with OS and TD	No role for the polymorphisms SOD2Val16Ala, CAT-262C>T, and GPX1Pro200Leu was found in TD.
(16) Kang et al. [12]	Manganese superoxide dismutase gene Ala-9Val polymorphism might be related to the severity of abnormal involuntary movements in Korean schizophrenic patients	Whether the *MnSOD* gene and Ala-9Val polymorphism is associated with TD	The study does not claim that the *MnSOD* gene Ala-9Val polymorphism was associated with TD.

## Abbreviations

TD: Tardive dyskinesia
SOD: Superoxide dismutase
SCZ: Schizophrenia
CuZnSOD: Copper-zinc superoxide dismutase
AP: Antipsychotic
ROS: Reactive oxygen species
BD: Bipolar disorder
OS: Oxidative stress
PCR: Polymerase chain reaction.

## Additional Points

*Key Points*. Studies found an association between TD and various polymorphisms. TD-related genetic polymorphisms include MnSOD -9val and DRD3 9ser polymorphisms. TD risk is associated with altered SOD levels.
